# Evidence‐based medicine and private clinics in Russia: Unlikely co‐production of good care and profit‐making

**DOI:** 10.1111/maq.70016

**Published:** 2025-07-28

**Authors:** Masha Denisova, Olga Zvonareva, Klasien Horstman

**Affiliations:** ^1^ Faculty of Health, Medicine, Life Sciences Department of Health, Ethics and Society, Health Inequities and Societal Participation (HISP), Care and Public Health Research Institute (CAPHRI) Maastricht University Maastricht the Netherlands

**Keywords:** boundary work, co‐production of care, evidence‐based medicine, private healthcare, Russia

## Abstract

Critical social science research demonstrates that evidence‐based medicine (EBM) emerged through its proponents’ deliberate efforts to defend EBM's knowledge production methods as credible and independent of commercial interests. In the present study, we expand this discussion by showing how EBM is co‐produced with profit‐making within the context of private clinics in Russia. Drawing on the ethnography of three private clinics in Russia, we explore how they strategically articulate EBM ideals to demarcate the boundaries between good and bad medical practices. We identified four forms of boundary work that private clinics perform to define their epistemic culture as different from those applying poor quality evidence, providing harmful prescriptions, over‐relying on clinical experience, and practicing a top‐down approach in patient relations. We discuss how, in the Russian healthcare context, EBM, instead of becoming the opposite of commerce, has become interwoven with and even dependent on private healthcare.

## INTRODUCTION

This research began with the empirical observation of how private clinics in large Russian cities started promoting their services as guided by principles of evidence‐based medicine (EBM). The term EBM, known in Russian as “dokazatel'naya meditsina,” has only recently begun gaining popularity among Russian patients and physicians. Compared to Western healthcare systems where EBM is a dominant medical paradigm (Guyatt et al., 2002), in Russia, EBM has only been partially integrated into the healthcare system (Borozdina, 2023). Recently, some private clinics actively promote EBM as a guarantee of better‐quality care and a patient‐centered approach that is worth paying for. By appealing to the principles of EBM, these clinics engage in epistemic disputes about what constitutes reliable evidence and good care. In this article, we follow these clinics to understand how this ideal of EBM became so closely intertwined with profit‐making interests.

As defined by its founding fathers, EBM involves “the conscientious, explicit, and judicious use of current best evidence in making decisions about the care of individual patients” (Sackett et al., 1996, 71). This best scientific evidence is conceived as largely made of the results of randomized controlled trials (RCTs) and their meta‐analyses, which are widely expected to produce universally applicable clinical evidence. However, this universalizing rhetoric has long been placed under critical scrutiny. Social science scholars, including those from science and technology studies (STS), have argued that the rise of EBM as a new “medical paradigm” served historically and culturally specific purposes within specific national settings, such as the United States and the United Kingdom (Carpenter, 2010; Marks, 1997; Timmermans & Berg, 2003). They argued that EBM is not, as its proponents claim, the mere application of objective scientific evidence to achieve superior clinical outcomes. Rather, it is a legitimizing strategy to out‐compete other approaches to medical knowledge production and conceptions of good care. In the contexts where EBM originated—sites such as the United States, where trust in research on medical technologies and pharmaceuticals has been compromised by the profit motives of the pharmaceutical companies (Carpenter, 2010; Marks, 1997)—EBM arose to distinguish science from commercial interests.

The story of “EBM‐oriented” private clinics in Russia contrasts with findings from existing studies. It shows how, under specific circumstances, profit‐oriented motives and good care can mutually constitute each other. Based on a qualitative study of three private clinics in Russian metropolitan cities,^1^ we demonstrate how they mobilize EBM as a particular way of knowing to legitimize their private medical practice. We scrutinize how, in the pursuit to differentiate their “evidence‐based” expertise from that of other healthcare organizations, these clinics continue to build and maintain boundaries between good and bad medical practice. We argue that, far from being a mere marketing slogan, EBM becomes a means of promoting better healthcare in the challenging healthcare environment, characterized by both monopolistic state control over production and use of medical knowledge and healthcare consumerism (Temkina & Rivkin‐Fish, 2020).

In exploring these issues, we draw on STS and medical anthropology studies focusing on the cultural and political situatedness of EBM. In addition, we employ the concept of boundary work (Gieryn, 1983) to understand how private clinics in Russia produce a particular conception of EBM and how this conception is enacted. Specifically, we are interested in how, by doing boundary work, private clinics balance their commercial interests and ideals of good care, while protecting the legitimacy of their medical practice. After presenting our methodology, we examine four forms of boundary work that private clinics utilize when differentiating their epistemic culture from other healthcare practices prominent in Russian healthcare. We conclude with a discussion on how the ideal of good care and commercial interests can be co‐produced, rather than seen through the prism of hostile worlds.

## SITUATED BOUNDARY WORK TO CONSTRUCT EBM

While EBM is often portrayed as a global movement to streamline medical practice, emphasizing the universal value of RCT‐based evidence and standardized nature of clinical guidelines (Guyatt et al., 2002), STS scholars and medical anthropologists have shown how EBM is situated in the local cultural and political contexts. Scholars examining the global economy of RCTs have demonstrated that the meanings and consequences of clinical trials are being reshaped by local actors and contingent practices (Merz, 2021; Petryna, 2009; Sariola & Simpson, 2011). The global expansion of industry‐sponsored clinical trials to lower‐income countries, including those in Eastern Europe and Latin America, has reconfigured local therapeutic landscapes, introducing new research and care opportunities while simultaneously bringing new risks to treatment‐naive populations and local public health systems (Cooper, 2008; Petryna, 2009; Zvonareva et al., 2017). Research on clinical practice has shown that uniformity of standards does not automatically result in coherent and predictable medical practices; rather, medical professionals continuously assess and adapt these standards to patient needs and local settings (Engel & Zeiss, 2014; Timmermans & Berg, 2003; Timmermans & Epstein, 2010; Zuiderent‐Jerak, 2007). Overall, contrary to typical perceptions, EBM is being continually reconfigured together with local contexts.

Importantly, this situatedness also concerns the ideal of good care embedded in the EBM philosophy. While the EBM movement typically considers good patient care to be derived from the practical application of clinical research evidence (Sackett et al., 1996), STS scholars have shown that the conception of good care is being continuously redefined within relational practices. Studying telecare in the Netherlands, Pols (2015) proposed analyzing care as a situated practice, embodying “different and sometimes conflicting notions of what is good care *within* care practices” (82). This approach allows for the exploration of ethics in practice: how people understand goodness in a specific situation and justify their practices as good in front of others (Heimer, 2013; Pols, 2015; Ziewitz, 2019). In healthcare settings, where commercial interests are historically at odds with the ideals of good care, defending one's integrity becomes crucial for businesses and industries to sustain their market presence (Martin, 2006; Sismondo & Chloubova, 2016).

In this article, we will expand on this scholarship by moving away from the Euro‐American contexts to the lesser studied political and cultural landscape of Russia. By following the work of three private clinics, we observe how they construct EBM by promoting their healthcare services as high quality and hence worth paying for. To understand how private clinics differentiate their EBM‐oriented care from other medical practices, we utilize the concept of boundary work.

### Boundary work

STS scholars have demonstrated that the establishment of EBM as a new medical paradigm has relied on practices of differentiation between credible and noncredible knowledge production methods (Carpenter, 2010; Marks, 1997; Timmermans & Berg, 2003). Gieryn (1983) introduced the concept of “boundary work” to understand how scientists employ different rhetorical tools to differentiate science from nonscience in the struggles over epistemic authority. He argued that “scientificity” is not an inherent characteristic of particular methods but is situated in a particular time and place (Gieryn, 1999). Importantly, this boundary‐making process is inherently political, as it renders what scientific methods are deemed legitimate, and restructures professional hierarchies and expertise.

Even institutionalized categories and boundaries, such as “scientific evidence” or “biomedicine,” are being subjected to reformulation and renegotiation through boundary work (Bal, 2005; Campbell, 2011; Petersen et al., 2015). For example, Ma and Lynch (2014) studied the controversy surrounding the appropriateness of adopting Western medical technologies, such as CT scanners, within traditional Korean medicine. They demonstrated that the distinctions between “Korean” and “Western” medicine were not based on their inherent ideological differences but were discursively constructed and negotiated, illustrating the lack of a stable epistemic boundary between the two medical paradigms. Mizrachi and Shuval (2005) explored the boundary work between biomedical physicians and their colleagues practicing “alternative medicine” at a Tel Aviv hospital. It showed that the distinctions between the two medical approaches and the epistemic authority of biomedicine were strictly established only at a formal level, while in daily interactions between the two groups of physicians, the boundary was less clear‐cut, allowing for the informal recognition of some alternative medical practices as helpful for patients. Drawing on these studies, we examine negotiations involved in private clinics’ boundary work when it comes to applying EBM ideals in practice.

## RUSSIAN HEALTHCARE SYSTEM AND PRIVATE CLINICS

Since the collapse of the USSR in 1991, Russian healthcare has undergone several major changes, informed by the neoliberal ideology. To increase productivity and competition in healthcare while keeping the citizens’ rights to free healthcare embedded in the Soviet health system, reformers introduced the compulsory medical insurance (CMI) program (Cook, 2017; Twigg, 1998). It entailed the creation of local CMI funds that redistribute financial resources among healthcare organizations and insurance companies that ensure the cost‐effectiveness of healthcare services. The healthcare system was also subjected to rapid marketization: public hospitals were allowed to provide paid medical services, and private clinics emerged. This transition resulted in substantial challenges, including intensified healthcare inequalities and shortages. The CMI program was poorly financed and inconsistently implemented, forcing many public hospitals to offer paid healthcare services (Cook, 2017). Russian physicians were suddenly confronted with a radically changed landscape and struggled to navigate new healthcare regulations, emergent consumer demands, and challenging working conditions. Research indicates that emerging consumer relations challenged the paternalistic style of patient‐physician relations dominant in the USSR, forcing physicians to adapt to new consumer expectations while working under precarious conditions (Temkina & Rivkin‐Fish, 2020).

In terms of knowledge, Russian healthcare has experienced an influx of new epistemic ideals and practices, such as EBM and multinational clinical trials, and attempts to restructure medical education programs. With the Iron Curtain lifted, segments of Russian physicians welcomed the ideology of EBM. Sociologist Anna Geltzer (2009) observed that these physicians used EBM as a rhetorical tool in their struggles to redefine their professional identities as distinct from the Soviet past and in relation to the international medical community. However, practical implementation of EBM in the healthcare system was hardly possible due to the insufficient infrastructure, lack of appropriate medical training, and bureaucratic obstacles (Geltzer, 2009). Still, the Russian Ministry of Health attempted to improve the quality of medical care by introducing obligatory medical standards touted as evidence‐based. Produced solely by the Ministry, these standards were sometimes based on international clinical guidelines; however, their specific sources remain largely unknown. Research indicates these standards are often redundant or incomplete (Kamenshchikova, 2018). In addition to standards, since 2022, more comprehensive clinical recommendations have become a compulsory component of medical practice regulation. A recent sociological study identified divergence between the content of the medical standards and the clinical recommendations that require physicians to undertake different actions (Borozdina, 2023). Additionally, physicians at public hospitals frequently question the quality of Russian recommendations and standards and often informally turn to Western clinical guidelines in their daily practice (Borozdina, 2023).

It is in such circumstances that private clinics in large Russian cities began claiming to bring better medical care by following “EBM principles.” This is, perhaps, not so surprising considering that private healthcare organizations in Russia have been shown to have greater organizational flexibility to locally innovate medical care (Borozdina, 2018; Borozdina & Novkunskaya, 2020). Existing ethnographic research on private maternity care indicates that medical professionals working in private spaces often enjoy greater autonomy and better working conditions, allowing them to provide more personalized medical care (Borozdina, 2018; Temkina & Rivkin‐Fish, 2020).

Since their initial appearance and emergence in the 1990s, private clinics are now present in every large Russian city and account for approximately 30% of outpatient healthcare organizations (Federal State Statistics Service, 2021). Private clinics in Russia are free to regulate their prices for offered healthcare services that range greatly depending on the geographical location, the clinic's reputation, the physician's qualification, and other factors. However, some clinics offer specific services free‐of‐charge under the CMI program.^2^ Private clinics typically consist of outpatient healthcare facilities, with most simultaneously providing several types of specialized medical services similar to those in public hospitals (Shishkin et al., 2013). Private clinics strive to distinguish themselves from public healthcare organizations with polite and welcoming personnel, quick appointment scheduling, and well‐maintained equipment and facilities. Partly because of the good service and comfort, many patients perceive healthcare provided in private clinics as of higher quality compared to public healthcare organizations (Levada Center, 2016). At the same time, many others also criticize them for prioritizing profits over patient needs.

## METHODOLOGY

To investigate this specific interconnection between good care and profit‐making, we conducted an ethnographic study of *three* “EBM‐oriented” private clinics in two Russian metropolitan cities. These clinics enjoyed a good reputation among the cities’ residents and were mostly oriented toward middle‐class clientele.

The fieldwork was conducted by the first author MD and consisted of two rounds. It relied primarily on 26 in‐depth, semi‐structured interviews with the clinics’ staff, patients, and professional partners. These interviews were supplemented with situational observations of the clinic's activities and an analysis of digital materials, such as the clinics’ websites, social media, and recordings of public talks delivered by the clinics’ physicians and of a conference for private clinics. Due to the COVID‐19 restrictions and, later, Russia's war in Ukraine, 18 out of 26 interviews were conducted online, while the opportunities for ethnographic observations were drastically limited. Conducting research during the unfolding pandemic in the large city required MD to stay alert to risks. By June 2021, the COVID‐19 cases were nearing their peak, and sports centers had been repurposed as temporary hospitals. Yet, city life carried on as usual: restaurants, cinemas, and shopping malls stayed fully open, while in public spaces, people rarely wore masks and almost never kept their distance. MD had to regularly get tested, avoid populated public spaces, and find alternatives to face‐to‐face interviews whenever possible.

During the first round of fieldwork in May–July 2021, MD traveled to Russia and approached two private clinics located in City A. Both clinics offered commercial healthcare services, with a few providing free‐of‐charge services via the CMI program. The first clinic was *a large multi‐specialty center*, offering services in general practice, gynecology, neurology, and more, with branches in several Russian cities. At the time of the study, the clinic had recently undergone significant reorganization, with new management aiming to make the clinic more EBM oriented. For this reason, the management introduced a research department responsible for the dissemination of new medical knowledge within the clinic and quality control. Nine interviews in the multi‐specialty clinic were conducted with a clinic commercial director (1), a marketing agent (1), physicians (4), and members of the research department (3). The second clinic was a *small oncology clinic* run by a close community of oncologists that had opened a few years prior to the study. It focused mainly on consultations, along with diagnostic and chemotherapy services. The clinic provided patients with on‐site healthcare services and referred them to the clinic's informal partners in different healthcare organizations for other types of medical services. Eleven interviews were conducted with the clinic's director (2), a head manager (1), a marketing agent (1), oncologists (3), and the clinic's informal partners, including oncologists at a federal hospital (2), a mammologist^3^ at a public hospital (who was also working in a private clinic) (1), and a urologist at a private clinic in a neighboring region (1).

During the fieldwork, the research participants often mentioned one private clinic as a pioneer in the EBM movement among the Russian private clinics. MD approached this clinic during the second round of online fieldwork in May–September 2022. It was a *small, multi‐specialty clinic* located in City B and operated solely on a commercial basis. It provided different outpatient healthcare services with the possibility of arranging inpatient care at other healthcare organizations—partners of the clinic. This clinic was established a few years before the study, evolving from a small community of physicians who shared similar knowledge ideals. MD conducted six online interviews with a director of the clinic (plus one follow‐up interview), oncologists (2), and patients (2). While we planned to do more interviews with personnel and patients, we decided not to, as the clinic was experiencing an increased workload due to the prevailing uncertainty and loss of clientele and international partners as consequences of Russia's war in Ukraine. Nevertheless, we continued following the clinic's public activity.

To analyze the collected data, we conducted a thematic analysis (Green & Thorogood, 2018). Because the interviews involved sensitive information, we anonymized the clinics and the research participants’ identifiers, replacing participant names with pseudonyms and omitting specific city names. Oral informed consent was obtained from the participants to ensure that there was no identifiable link between the identities of the research participants and the data they provided. The study received ethical clearance from Research Ethics Committee of Faculty of Health, Medicine & Life Sciences, Maastricht university (FHML‐REC). The approval number is FHML‐REC/2021/067​.

## FINDINGS

Members of the private clinics put much effort into differentiating their healthcare services from those provided by other healthcare organizations. They appealed to the principles of EBM to show and defend their image as clinics providing high‐quality and ethically sound medical care. By constructing their practices around EBM, the research participants were constantly engaged in four different forms of boundary work.

### Differentiating from poor‐quality evidence

When defining EBM, the research participants often differentiated “good quality” evidence produced via RCTs in the Western context from “poor quality” evidence produced in Russia. The clinic members and their partners expressed mistrust of Russian healthcare authorities and institutions. They were skeptical about medical statistics provided by the Ministry of Health, believing that some data were produced solely to gloss over health problems rather than to reflect the actual situation. Drawing on their personal experiences working at public hospitals, the physicians provided examples of how hospital‐based studies were heavily manipulated to meet the desired hypothesis and how hospitals falsified statistical data to signal improvements in the health status of the population. Some research participants further connected data manipulations with the lack of a well‐established research tradition in Russia, highlighting that many medical professionals do not understand how statistical methods operate and are poorly informed about the principles of research integrity.

In contrast, the RCT methodology, as developed in the West, was seen as superior. The research participants associated the methodology deployed in RCTs with doing “honest” research: transparent, strictly monitored, and independent from the will of the hospital or state.

[EBM] It's honestly proven medicine… (laughs) it's honest samples, it's honest databases, it's multicenter trials, it's randomized trials, so many conditions must be met. *(Oncologist at a federal hospital, partner of the small oncology clinic)*

[EBM] is medicine built on statistical data proving the effectiveness of medicines based on double‐blind randomized trials, not just simple ones. When you attend local conferences, you will see that medicines get prescribed based on a study involving only five patients, showing that it helped them. This sort of thing happens quite often. *(Urologist at a private clinic, partner of the small oncology clinic)*

Distrust in the procedures involved in producing clinical evidence in Russia fueled the research participants’ doubts about the quality of Russian medical standards. Physicians at private clinics primarily viewed these standards as administrative cost‐control mechanisms detached from the realities of medical practice, characterizing some of them as outdated and overly prescriptive. They also questioned the quality of recently emerging Russian clinical recommendations, arguing that while some were translations of international guidelines and hence of decent quality, they still were not fully up‐to‐date and sometimes referenced inferior‐quality medicines. Some research participants also expressed concerns about the obligatory character of these recommendations, which would prevent physicians from adjusting them to provide better patient care. Unsatisfied with local medical standards and clinical recommendations, private clinics have resorted to using guidelines and evidence produced elsewhere. For this purpose, they relied on RCT‐based international clinical guidelines and recently published results of clinical trials published in international journals.

To navigate with guidelines undergoing continuous updating and the frequent publication of new RCT results, private clinics used medical information systems, such as UpToDate or Algom. Both systems provide summaries of recent clinical guidelines and medical literature to support clinical decision‐making. While UpToDate has been widely used in different national contexts, Algom has been specifically adapted for the Russian healthcare context, meaning it includes Russian clinical recommendations and medicines typically available in Russia. Subscription to these information and support systems was rather expensive, with the private clinics’ management taking pride in providing their physicians with access to such evidence‐based knowledge and bringing their clinics closer to those “in the West.”

At the same time, to utilize these resources of good evidence, private clinics had to outmaneuver Russian healthcare regulations. Because Russian medical standards and recommendations have an obligatory status and must be applied to every patient with specific conditions, private clinics have to work around them. For example, physicians at the large multi‐specialty clinic would make a written prescription based on medical standards and orally communicate to patients any deviations from the written treatment plan, or they would provide additional recommendations to the main treatment plan, citing Western clinical guidelines. The members of the small oncology clinic shared that the clinic's medical documentation was rarely checked by healthcare authorities, which allowed them to deviate from blindly following the Russian medical standards. At the same time, when clinics participated in the CMI program, they had no choice but to adhere strictly to the Russian medical standards to qualify for reimbursement. In the CMI case, insurance companies thoroughly checked the clinics’ documentation, with any deviations from the standards leading to no reimbursement for private clinics. As some interlocutors emphasized, practicing “real” EBM was only possible in purely commercial settings:

When we work on a commercial basis and see in clinical recommendations—Russian, Western—that some treatment is effective for this particular patient and accepted by the world community, but this treatment schema is not included in the CMI rates…. Then, in principle, we have nothing to prevent us from using it. When we write about it on the website, we, of course, need to say in brackets that this works only on a commercial basis (chuckles). Because if a patient is treated under the CMI program and is unlucky in a way that a recommended treatment schema is not involved in the CMI, then we won't be able to do it. *(Director of the small oncology clinic)*

By maneuvering the application of Russian medical standards, private clinics created opportunities to employ Western clinical guidelines and recommendations that they believed to be of better quality than “outdated” and “poor‐quality” evidence produced by the Russian health authorities. To justify these deviations, private clinics mobilized the rhetoric of mistrust toward state control over the production of medical standards.

Distancing from authoritative state agendas and local ways of producing evidence helped physicians at private clinics to reaffirm their professional autonomy and medical expertise—a process that was observed in the studies on Soviet and post‐Soviet Russian medical professionals (Geltzer, 2009; Leykin & Rivkin‐Fish, 2022). This research has demonstrated that medical professionals in Soviet and early post‐Soviet periods negotiated their medical expertise and professional authority as distinct from explicit state agendas. In our study, we observed that efforts to reassert medical expertise and integrity were complicated by the clinics’ equal distrust of the market, specifically of other private clinics deemed overly profit‐driven.

### Differentiating from harmful prescription practices

In Russia, private clinics are often publicly criticized for over‐prescribing services and medications to earn additional profits. Aware of the private sector's poor reputation, the clinics we studied aimed to depict themselves as ethical by distancing themselves from other “greedy” private clinics. The research participants portrayed “greedy private clinics” as those operating under a manipulative “pricing strategy,” when the management of a private clinic specifies a certain target sum that patients are expected to spend in the clinic. The physicians at those clinics are then pressured to prescribe treatments and diagnostic procedures to ensure that the target is met, thus prioritizing earning profits for the clinic over patient needs. Our interviewees provided examples of how such clinics prescribe antibiotics or vacuum nasal cleaning to treat simple viral infections or promote complex checkups for young and healthy people.

To abstain from over‐prescription practices, private clinics’ staff relied on EBM as a tool guaranteeing only needed, high‐quality medical care:

What is EBM for a private clinic? A patient comes to me, and as a chemotherapist, I understand that at this stage, it's possible to operate on this patient. However, in the clinic's interest to make money, they want to administer chemotherapy. So, with this selfish motive, I would say, “Let's start with chemotherapy.” But that's not right. What's right is this: I understand that I'm a chemotherapist, but a patient should first go to the surgeon. They go to the surgeon first, and then come to me if there's something concerning in the histology. *(Senior oncologist, a small oncology clinic)*

In this quote, EBM is approached as an ethical principle that prevents physicians from over‐prescribing unnecessary medical procedures and medications for the sake of earning extra profits. By appealing to EBM, private clinics downplayed their commercial motives and portrayed themselves as “honest” businesses. This distinction enabled clinics to portray themselves as morally superior to other private clinics and was considered a great market advantage. A director of the small multi‐specialty clinic emphasized that being an EBM‐driven clinic involved refusing to prescribe unnecessary treatments, even when they were requested.

Along with over‐prescription, harmful practices included prescribing medications without proven efficacy, derogatorily called “fooflomicine” (derived from the Russian jargon “fooflo,” meaning “junk,” and “micine” for “medicines”). From the first interviews, we learned that fooflomicines were well‐known adversaries in EBM circles. The physicians perceived them as medicines that have minimal to no proven efficacy: either existing RCTs demonstrate their lack of effectiveness, or no trials whatsoever have been undertaken concerning them. The physicians criticized the widespread usage of fooflomicines in medical practice in both public and private settings. By appealing to EBM, private clinics strove to avoid prescriptions of ineffective medications and educated their patients about their misuse:

The popularization of EBM resulted in people reading more about [the effectiveness] of medicines. For example, a child has a respiratory infection and is prescribed antibiotics. Why is that? This type of discussion has become more common. In Germany, there is one medication for treating a runny nose, while in Russia, there are 35 medications for the same purpose. So, people start to question the abundance of fooflomicines. *(Head of the research department, large multi‐specialty clinic)*

However, some physicians at private clinics found themselves having to compromise their ideals of good prescriptions in their day‐to‐day practice. Several physicians acknowledged that prescribing medications is not simply the mechanical task of excluding ineffective drugs. Rather, they saw it as a pragmatic practice requiring attention to the patients’ needs and the potential benefits of a medicine. For example, a neurologist at a multi‐specialty clinic shared that although he did not believe in the efficacy of fooflomicines, he still occasionally prescribed them to patients to gain their trust. He explained that older patients who were accustomed to the medicines popular in Soviet times expected him to prescribe glycine, an amino acid that promises to support the nervous system's functioning. In such cases, he prescribed it as a supplementary drug so that a patient would become more receptive to his prescriptions of other medications that he deemed effective. Oncologists at a small oncology clinic also did not prevent their patients from using “homeopathic” drugs if they agreed to adhere to the prescribed cancer treatment. Hence, physicians could tolerate the use of fooflomicines if they facilitated patient adherence to treatment without imposing direct harm. These findings resonate with Raikhel's (2010) research on placebo treatment for alcohol addiction in post‐Soviet Russia, where treatment efficiency was constructed not solely by its pharmacological effects but by its pragmatic aid for patient care.

When negotiating the boundaries between good and harmful prescription practices, private clinics strived to portray themselves as an “ethical” healthcare provider in the healthcare market populated with “greedy” private clinics. At the same time, when the ideals of good prescriptions could not be fully realized in practice, the research participants evoked a category of the patient good (i.e., well‐being). Prescribing unnecessary and ineffective treatments to ensure patient trust and their adherence to treatment was tolerated, provided that there are no reasons to expect harm, while using the same drugs solely for making extra profit was considered deceitful and harmful.

### Differentiating from overreliance on clinical experience

When discussing clinical decision‐making, the research participants distinguished between reliance on RCT‐based clinical guidelines and the experience of an individual practitioner. They believed that the best medical care could be achieved when a practitioner was primarily guided by knowledge from recent clinical research and not solely by clinical experience. Implicitly, they critiqued the dominant professional hierarchies in Russian healthcare. The physicians to whom we talked recounted multiple instances in their medical careers when practitioners of higher professional status dictated the procedures for practicing medical care without substantiating such claims with statistical evidence. According to them, mandates based on a single person's opinion could result in suboptimal or even inadequate diagnostics and treatments. As a surgeon at a public hospital and a partner of the small oncology clinic recalled:

The attending physician was operating when the question arose whether the patient needed a [surgical] drain inserted. The older generation says, “You need to do it because you will sleep peacefully.” I ask, “In what sense?” He replied, “This way you will see, if blood flows through, then the patient needs to be taken urgently for reassessment and reoperation.” I respond, “What if the drain clogs? Sticks together? How will I know then?” <…> It's not only me; my younger colleagues share these same concerns. So, you start looking for an answer to the question, most often using all these databases <…> And here the word “EBM” appears because your decision is supported not by someone's experience or professional status <…> but by the fact that this study was performed on thousands of patients, which shows that if you have such surgery, then you cannot put a drain, because the probability that you will see blood in it is only, for example, 0.1%. It is a small number, so it makes no sense to insert it.

In this quote, she described a typical situation at a public hospital when the clinical experience of an established physician directed a course of action. To question such an approach to decision‐making means challenging the authority of a senior professional and the hospital's established order—a behavior that is not easily tolerated in public healthcare settings where strict subordination is the norm (Kuhlmann et al., 2019; Litvina et al., 2019). An oncologist‐chemotherapist at a small oncology clinic shared that one of the reasons she left her work at a public hospital was her struggle to correct the hospital's established routines.

In contrast, the research participants emphasized their reliance on scientific evidence, preferably from RCTs, when making decisions about diagnosis and treatment. Importantly, generational factors were evoked in this boundary‐making process. The interviewees widely perceived the older physicians as “stuck” in the Soviet‐style education and professional hierarchies, which made them resistant to new medical approaches like EBM. These physicians were often portrayed as blindly following personal experience of treating patients using the same methods that were learned at their medical university decades ago. In contrast, the “new generation” of physicians, who have a greater chance of being exposed to the ideas of EBM in their education, were considered to be more open to new medical approaches. Notably, most of the physicians we interviewed were young professionals in their 30s to early 40s, with some having courses in medical statistics in their professional education or even attending specific educational programs inspired by the EBM ideology. Many among them believed that by relying on recent clinical research and guidelines, they would not only bring better medical care but also achieve greater professional autonomy. Ultimately, they considered EBM a means of democratizing medicine in nondemocratic healthcare and society.

And what distinguishes the approach of EBM, in general, in contemporary medicine from the one that is still, unfortunately, prevalent in Russia? Here, there is no pressure from authority. The right answer does not belong to those with higher scientific status; the right answer belongs to those who have data, research results. The latter does not tolerate such authoritarianism. *(Oncologist, a small multi‐specialty clinic)*

At the same time, in their clinical practice, the physicians at private clinics balanced the use of scientific evidence with reliance on their own judgment. They provided cases in which patients had complex medical conditions or suffered from additional diseases requiring modifications to standardized treatment protocols. In such instances, deviating from strict adherence to clinical guidelines was deemed necessary for the benefit of the patient:

It is believed that every prescription you make must be based on a clinical trial. This is all cool… The first two or three lines of treatment are all good. But what if a patient feels unwell and has formally exhausted all his evidence‐based options? This is when a discussion arises about what else can be done, how to help the patient, for example, to make a non‐standard solution. *(Senior oncologist, a small oncology clinic)*

Overall, the interviewees embraced EBM as an approach that places most epistemic authority in soundly designed scientific research, rather than in the experience of credentialed physicians. They perceived the indisputable authority of high‐status physicians and the habit of over‐relying on one's personal experience as relics of the past. Similar to the physicians studied by Geltzer (2009), our research participants mobilized EBM to redefine their professional identity as distinct from local and “outdated” knowledge practices. By prioritizing scientific evidence in clinical decision‐making, private clinics rhetorically positioned themselves as representatives of the future of the medical profession and hence claimed their knowledge superiority over other healthcare organizations.

### Differentiating from top‐down patient relations

The fourth distinction research participants made was between partnership‐based and top‐down approaches to patient relations. For them, EBM entailed not only shifts in the professional hierarchies but also in the patient‐physician relations.

Russian healthcare is known for its hierarchical and patronizing doctor–patient relationships, which are commonly accompanied by assembly‐line healthcare services and a rude communication style (Temkina et al., 2021). According to the interviewees, in this environment, patients are assigned the role of passive recipients of healthcare services, whose reasoning and treatment preferences are often completely neglected. Patients are often seen as unknowledgeable subjects, while physicians are seen as authoritative figures with the sole power to determine treatment paths. The private clinics’ members problematized this paternalistic approach to patients, highlighting the complete failure to recognize patient agency within the Russian healthcare system. For them, achieving good care, even when it was based on evidence, was not possible without promoting a more respectful and attentive approach to patients’ needs:

You can call yourself as evidence‐based as you like, but if you haven't explained it to the patient, if you haven't clarified it for the patient, then all that evidence isn't worth a penny [“grosh zena,” in Russian]. *(Head of the research department, large multi‐specialty clinic)*

The private clinics’ members emphasized that their focus on “patient centeredness” set them apart from other healthcare organizations. This patient‐oriented approach, as they portrayed it, revolved around cultivating trusting relationships with patients and was based on mutual respect and shared decision‐making. Within these interactions, patients’ needs and experiences were prioritized, and active involvement in treatment decisions was encouraged. Both managers and physicians explained that the organizational setup of private clinics allowed for a more personalized and continuous approach compared to the standard treatment routes in public hospitals. For example, upon entering a private clinic, patients could anticipate having an assigned physician overseeing and guiding their entire medical pathway. The extended appointment times, ranging from 30 min to 1.5 h, allowed the clinics’ physicians to delve into the nuances of the medical cases and better accommodate patient needs.

Simultaneously, the private clinics’ members acknowledged that the patient‐centered approach was a rather recent arrival to Russian healthcare and, at times, was met with resistance. Several physicians noted that, at times, patients considered shared decision‐making as irrelevant or even preventing the provision of good care. Accustomed to rigid power hierarchies in patient–physician relations, some patients, particularly from older generations, believed that a competent doctor should autonomously make decisions. Failing to do so was seen as weakness or inexperience in the patient's eyes. Other patients might also feel uncomfortable in the newly prescribed role of active participant:

If the patient is open, then decisions should be made together, but some patients are not capable of that. <…> Sometimes a man comes with his wife because he's accustomed to listening to her opinion in everyday matters, and it's easier for him to get information from her rather than from the doctor. In other words, he can't make the decision himself; he needs the support of a close person. And for such a patient, it's impossible to provide two options and say, “You can choose: either we perform the surgery now, or we keep you under active observation for six months.” And a person like this will say, “I don't know. How can I choose? I'm not a doctor!” And such people bring a relative with them and wait for their answer. *(Oncologist, a small multi‐specialty clinic)*

Navigating this new approach to patient relations sometimes was challenging for medical professionals. For example, some physicians at the small oncology clinic struggled to uphold a professional distance from worried patients trying to contact them day and night for additional support and reassurance. The large multi‐specialty clinics that have recently undergone reorganization introduced a new educational program for patient communication that was compulsory for all physicians. According to the research department responsible for its implementation, many physicians found it challenging to adapt to new communication standards, and some even quit. The introduction of such programs, combined with a willingness to bear staff losses, signaled the clinics’ commitment to patient‐centered care.

Private clinics put much effort into promoting new norms for transparent and respectful communication with patients, including listening to their needs and complaints, informing them about treatment options and potential consequences, and taking into consideration their preferences. They strategically cultivated the image of their physicians as attentive and encouraging cooperation rather than domineering over patients. In short, clinics’ members believed in patient‐centered care based on shared decision‐making and responsibility. These findings are intriguing and contrast with the existing studies on private healthcare in Russia. In their ethnographic research on the commercialization of maternity care over the past 30 years, Temkina and Rivkin‐Fish (2020) demonstrated a failure of consumer relations to facilitate patient empowerment. Despite patients having greater choice, they still occupy a subordinate position in clinical decision‐making, with physicians maintaining an authoritative role (Temkina & Rivkin‐Fish, 2020). In contrast, the private clinics studied here strived to recognize patients as partners, and not only consumers. By claiming to depart from rigid hierarchical relations between physicians and patients, private clinics drew yet another boundary between themselves and other healthcare organizations.

## CONCLUSION

Several scholars have studied the cultural and political situatedness of EBM and demonstrated that with the rise of EBM in Euro‐American contexts, credible knowledge became associated with the independence of commercial interests (Carpenter, 2010; Marks, 1997). In the present study, we have sought to expand this discussion by showing how, in the context of Russia, EBM is being co‐produced with profit‐making. While profit‐oriented motives are usually assumed to corrupt good knowledge, in our study it became clear that, in some cases, they can mutually constitute each other. In the authoritative setting of Russian healthcare, private clinics can become protected organizational spaces allowing for greater professional autonomy and innovations in medical care.

By following the work of EBM‐oriented private clinics in Russia, we analyzed the interactional production between what counts as good evidence‐based care and good business. For private clinics operating in a competitive market, it is an effective marketing strategy to present themselves as providing fair, high‐quality healthcare services and prioritizing patients’ needs. Positioning themselves as EBM‐oriented allowed private clinics to carve out a new niche in a healthcare market, which our interviewees were aware of. At the same time, while constructing the meanings of EBM, private clinics were continuously engaged in building and maintaining boundaries between good and bad medical practices. Ultimately, they strived to define their epistemic culture and differentiate it from those applying poor‐quality evidence, providing harmful prescriptions, over‐relying on clinical experience, and practicing a top–down approach in patient relations. Through this boundary‐making process, members of private clinics justified to themselves and others the co‐existence of seemingly opposing logics of good care and commerce.

In this differentiation process, private clinics had to constantly balance the ideals of EBM with the realities of Russian healthcare. Our findings align with previous research on Russian medical professionals in public healthcare that demonstrated that despite multiple restrictions, physicians still find informal ways to exercise professional autonomy in applying the knowledge they trust (Borozdina, 2023; Borozdina & Novkunskaya, 2020). By relying on the international clinical guidelines and protocols as an alternative to outdated and authoritative Russian medical standards, private clinics’ members aimed to legitimize the integrity of their medical practice. At the same time, our analysis showed that private clinics had to continuously maneuver between the application of the international guidelines and compliance with Russian medical standards, and between avoiding prescriptions of ineffective medications and making patients trust their expertise. In doing so, they are taking professional risks. This often led to the situations in which medical professionals had to compromise certain EBM principles, such as transparency or adherence to the best evidence, in favor of upholding other aspects of EBM they deemed more important. To normalize for themselves and others these deviations from the ideal of EBM, they referred to the “patient good.” According to Pols (2015), goodness within care practice is continuously “contested by comparing it to alternative practices with different notions of good care” (91). For private clinics in the Russian healthcare landscape, patient good was formulated by contrasting it with harmful prescription practices and the top‐down approach to patient relations prevalent in other healthcare organizations.

It is worth acknowledging that, in the context of the Russian healthcare landscape, inequality is an urgent problem. The private clinics studied here present the case of exclusive healthcare spaces possessing sufficient financial, managerial, and social resources and a deep understanding of the intricacies of Russian healthcare regulations. Hence, they were able to provide their physicians with enough organizational support and security to practice EBM. Unlike physicians studied by Geltzer (2009) in the early 2000s, for whom EBM served as a discursive tool only, private clinics’ members in the current study found ways to translate their ideal of good care into real practices. This should not be surprising, considering that physicians at Russian public hospitals often find themselves in vulnerable positions and lack resources (Litvina et al., 2019; Shishkin, 2018). Thus, the success of the boundary work (Pereira, 2019) can also be understood by the relative privileges of private clinics. As ironically noted by the clinic's representative themselves, EBM in Russia became possible only in private and secured settings.

## CONFLICT OF INTEREST STATEMENT

The authors declare no conflicts of interest.

## ETHICS STATEMENT

This study received ethical approval from the Ethics Review Committee in the Faculty of Health, Medicine and Life Sciences at Maastricht University, the number of approval: FHML‐REC/2021/067. All research participants were informed about the study and gave their oral consent or signed a consent form before the interviews. The participants were informed of their rights to withdraw from the study at any time without any consequences. Confidentiality and anonymization were assured as a safeguard against any harm to the participants.

## ENDNOTES

1 The names of the cities were anonymized.

2 Healthcare services offered free of charge can include fertility treatment, specific chemotherapy schemas, trauma surgery, neurology, and ophthalmology services, among others.

3 In English, this profession is commonly known as a radiologist.

## References

[maq70016-bib-0001] Bal, Roland. 2005. “How to Kill With a Ballpoint: Credibility in Dutch Forensic Science.” Science, Technology, & Human Values 30(1): 52–75. 10.1177/0162243904270722

[maq70016-bib-0046] Borozdina, Ekaterina . 2018. “Introducing ‘Natural’ Childbirth in Russian Hospitals. Midwives' Institutional Work.” In Health, Technologies, and Politics in Post‐Soviet Settings: Navigating Uncertainties, 145–171. Cham: Springer International Publishing.

[maq70016-bib-0002] Borozdina, Ekaterina. 2023. “Evidence‐Based Medicine and Physicians' Institutional Agency in Russian Clinical Settings.” Critical Public Health 33(4): 409–420. 10.1080/09581596.2023.2180608

[maq70016-bib-0003] Borozdina, Ekaterina , and Anastasiia Novkunskaya . 2020. “Patient‐Centered Care in Russian Maternity Hospitals: Introducing a New Approach Through Professionals' Agency.” Health 26(2): 200–220. 10.1177/1363459320925871 32515662

[maq70016-bib-0004] Campbell, Patricia. 2011. “Boundaries and Risk: Media Framing of Assisted Reproductive Technologies and Older Mothers.” Social Science & Medicine 72(2): 265–272. 10.1016/j.socscimed.2010.10.028. 21131118

[maq70016-bib-0005] Carpenter, Daniel. 2010. Reputation and Power: Organizational Image and Pharmaceutical Regulation at the FDA. Princeton, NJ: Princeton University Press.

[maq70016-bib-0006] Cook, Linda J. 2017. “Constraints on Universal Health Care in the Russian Federation: Inequality, Informality and the Failures of Mandatory Health Insurance Reforms.” In Towards Universal Health Care in Emerging Economies, edited by Ilcheong Yi , 269–296. London: Palgrave Macmillan UK. 10.1057/978-1-137-53377-7_10

[maq70016-bib-0007] Cooper, Melinda. 2008. “Experimental Labour—Offshoring Clinical Trials to China.” East Asian Science, Technology and Society: An International Journal 2(1): 73–92. 10.1215/s12280-008-9040-y

[maq70016-bib-0008] Engel, Nora , and Ragna Zeiss . 2014. “Situating Standards in Practices: Multi Drug‐Resistant Tuberculosis Treatment in India.” Science as Culture 23(2): 201–225. 10.1080/09505431.2013.837875

[maq70016-bib-0009] Federal State Statistics Service . 2021. "Zdravoohranenie v Rossii. Statisticheskij Sbornik" ["Healthcare in Russia. Statistical Compendium"]. Moscow: Rosstat. https://rosstat.gov.ru/storage/mediabank/Zdravoohran‐2021.pdf

[maq70016-bib-0011] Geltzer, Anna. 2009. “When the Standards Aren't Standard: Evidence‐Based Medicine in the Russian Context.” Social Science & Medicine 68(3): 526–532. 10.1016/j.socscimed.2008.10.029 19036489

[maq70016-bib-0012] Gieryn, Thomas F. 1983. “Boundary‐Work and the Demarcation of Science From Non‐Science: Strains and Interests in Professional Ideologies of Scientists.” American Sociological Review 48(6): 781. 10.2307/2095325

[maq70016-bib-0013] Gieryn, Thomas F. 1999. Cultural Boundaries of Science: Credibility on the Line. Chicago, IL: The University of Chicago Press.

[maq70016-bib-0014] Green, Judith , and Nicki Thorogood . 2018. Qualitative Methods for Health Research. London: SAGE Publications.

[maq70016-bib-0015] Guyatt, Gordon H. , Brian Haynes , Roman Jaeschke , Deborah Cook , Trish Greenhalgh , Maureen Meade , Leila Green , C. David Naylor , Mark Wilson , Finlay McAlister , and W. Scott Richardson . 2002. “Introduction: The Philosophy of Evidence‐Based Medicine.” In Users' Guides to the Medical Literature: A Manual for Evidence‐Based Clinical Practice, edited by Gordon Guyatt and Drummond Rennie , 3–12. Chicago, IL: AMA Press.

[maq70016-bib-0016] Heimer, Carol A. 2013. “‘Wicked’ Ethics: Compliance Work and the Practice of Ethics in HIV Research.” Social Science & Medicine 98: 371–378. 10.1016/j.socscimed.2012.10.030 23312301

[maq70016-bib-0017] Kamenshchikova, Alena. 2018. “Medico‐Economic Standards in Russia: Balancing Legal Requirements and Patients Needs.” In Health, Technologies, and Politics in Post‐Soviet Settings, edited by Olga Zvonareva , Evgeniya Popova , and Klasien Horstman , 117–142. Cham: Palgrave Macmillan.

[maq70016-bib-0018] Kuhlmann, Ellen , Sergey Shishkin , Erica Richardson , Igor Ivanov , Oleg Shvabskii , Ildar Minulin , Aleksandra Shcheblykina , Anna Kontsevaya , Katie Bates , and Martin McKee . 2019. “Understanding the Role of Physicians Within the Managerial Structure of Russian Hospitals.” Health Policy 123(8): 773–781. 10.1016/j.healthpol.2019.05.020 31200948

[maq70016-bib-0019] Levada Center . 2016. "Otchet “Protivostoyaniye logik: Vrach, patsiyent i vlast' v usloviyakh reformirovaniya sistemy zdravookhraneniya” [Report “Clash of Logics: Doctor, Patient, and Authority in the Context of Healthcare System Reform”]. Moscow: Levada Center, accessed November 20, 2024. https://www.levada.ru/2016/05/05/protivostoyanie‐logik‐vrach‐patsient‐i‐vlast‐v‐usloviyah‐reformirovaniya‐sistemy‐zdravoohraneniya/

[maq70016-bib-0020] Leykin, Inna , and Michele Rivkin‐Fish . 2022. “Politicized Demography and Biomedical Authority in Post‐Soviet Russia.” Medical Anthropology 41(6–7): 702–717. 34709964 10.1080/01459740.2021.1987897

[maq70016-bib-0021] Litvina, Daria , Anastasia Novkunskaya , and Anna Temkina . 2019. “Multiple Vulnerabilities in Medical Settings: Invisible Suffering of Doctors.” Societies 10(1): 5. 10.3390/soc10010005

[maq70016-bib-0022] Ma, Eunjeong , and Michael Lynch . 2014. “Constructing the East–West Boundary: The Contested Place of a Modern Imaging Technology in South Korea's Dual Medical System.” Science, Technology, & Human Values 39(5): 639–665. 10.1177/0162243914526460

[maq70016-bib-0023] Marks, Harry M. 1997. The Progress of Experiment: Science and Therapeutic Reform in the United States, 1900–1990. Cambridge, MA: Cambridge University Press. 10.1136/bmj.316.7137.1101a PMC1112925 9552934

[maq70016-bib-0024] Martin, Emily. 2006. “Pharmaceutical Virtue.” Culture, Medicine and Psychiatry 30(2): 157–174. 10.1007/s11013-006-9014-2 16835808

[maq70016-bib-0025] Merz, Sibille. 2021. “Global Trials, Local Bodies: Negotiating Difference and Sameness in Indian For‐Profit Clinical Trials.” Science, Technology, & Human Values 46(4): 882–905. 10.1177/0162243920963813

[maq70016-bib-0027] Mizrachi, Nissim , and Judith T. Shuval . 2005. “Between Formal and Enacted Policy: Changing the Contours of Boundaries.” Social Science & Medicine 60(7): 1649–1660. 10.1016/j.socscimed.2004.08.016 15652695

[maq70016-bib-0028] Pereira, Maria Do . 2019. “Boundary‐Work That Does Not Work: Social Inequalities and the Non‐Performativity of Scientific Boundary‐Work.” Science, Technology, & Human Values 44(2): 338–365. 10.1177/0162243918795043

[maq70016-bib-0029] Petersen, Alan , Claire Tanner , and Megan Munsie . 2015. “Between Hope and Evidence: How Community Advisors Demarcate the Boundary Between Legitimate and Illegitimate Stem Cell Treatments.” Health 19(2): 188–206. 10.1177/1363459314555240 25367895

[maq70016-bib-0030] Petryna, Adriana. 2009. When Experiments Travel: Clinical Trials and the Global Search for Human Subjects. Princeton, NJ: Princeton University Press.

[maq70016-bib-0031] Pols, Jeannette. 2015. “Towards an Empirical Ethics in Care: Relations With Technologies in Health Care.” Medicine, Health Care and Philosophy 18(1): 81–90. 10.1007/s11019-014-9582-9 25023945

[maq70016-bib-0032] Raikhel, Eugene. 2010. “Post‐Soviet Placebos: Epistemology and Authority in Russian Treatments for Alcoholism.” Culture, Medicine, and Psychiatry 34: 132–168. 19967435 10.1007/s11013-009-9163-1

[maq70016-bib-0033] Sackett, David L. , William M. C. Rosenberg , J. A. Muir Gray , R. Brian Haynes , and W. Scott Richardson . 1996. “Evidence Based Medicine: What It Is and What It Isn't: It's About Integrating Individual Clinical Expertise and the Best External Evidence.” BMJ 312(7023): 71–72. 8555924 10.1136/bmj.312.7023.71 PMC2349778

[maq70016-bib-0034] Sariola, Salla , and Bob Simpson . 2011. “Theorising the ‘Human Subject’ in Biomedical Research: International Clinical Trials and Bioethics Discourses in Contemporary Sri Lanka.” Social Science & Medicine 73(4): 515–521. 10.1016/j.socscimed.2010.11.024 21208703

[maq70016-bib-0035] Shishkin, Sergey V. , Elena G. Potapchik , and Elena Selezneva . 2013. “Private Health Care Sector in Russia: Present State and Development Prospects.” Voprosy Ekonomiki 4: 94–112.

[maq70016-bib-0036] Shishkin, Sergey. 2018. “Health Care.” In Russia: Strategy, Policy and Administration, edited by Irvin Studin , 229–239. London: Palgrave Macmillan UK.

[maq70016-bib-0037] Sismondo, Sergio , and Zdenka Chloubova . 2016. “‘You're Not Just a Paid Monkey Reading Slides’: How Key Opinion Leaders Explain and Justify Their Work.” BioSocieties 11(2): 199–219. 10.1057/biosoc.2015.32

[maq70016-bib-0038] Temkina, Anna , Daria Litvina , and Anastasia Novkunskaya . 2021. “Emotional Styles in Russian Maternity Hospitals: Juggling Between Khamstvo and Smiling.” Emotions and Society 3(1): 95–113.

[maq70016-bib-0039] Temkina, Anna , and Michele Rivkin‐Fish . 2020. “Creating Health Care Consumers: The Negotiation of Un/Official Payments, Power and Trust in Russian Maternity Care.” Social Theory & Health 18(4): 340–357. 10.1057/s41285-019-00110-3

[maq70016-bib-0040] Timmermans, Stefan , and Marc Berg . 2003. The Gold Standard: The Challenge of Evidence‐Based Medicine and Standardization in Health Care. Philadelphia, PA: Temple University Press.

[maq70016-bib-0041] Timmermans, Stefan , and Steven Epstein . 2010. “A World of Standards but Not a Standard World: Toward a Sociology of Standards and Standardization.” Annual Review of Sociology 36(1): 69–89. 10.1146/annurev.soc.012809.102629

[maq70016-bib-0042] Twigg, Judyth L. 1998. “Balancing the State and the Market: Russia's Adoption of Obligatory Medical Insurance.” Europe‐Asia Studies 50(4): 583–602. 10.1080/09668139808412555

[maq70016-bib-0043] Ziewitz, Malte. 2019. “Rethinking Gaming: The Ethical Work of Optimization in Web Search Engines.” Social Studies of Science 49(5): 707–731. 10.1177/0306312719865607 31387459

[maq70016-bib-0044] Zuiderent‐Jerak, Teun . 2007. “Preventing Implementation: Exploring Interventions With Standardization in Healthcare.” Science as Culture 16(3): 311–329. 10.1080/09505430701568719.

[maq70016-bib-0045] Zvonareva, Olga , Nora Engel , Natalia Kutishenko , and Klasien Horstman . 2017. “(Re)Configuring Research Value: International Commercial Clinical Trials in the Russian Federation.” BioSocieties 12(3): 392–414. 10.1057/biosoc.2016.11.

